# Acceptance of physical activity virtual reality games by residents of long-term care facilities: A qualitative study

**DOI:** 10.1371/journal.pone.0305865

**Published:** 2024-06-25

**Authors:** Marjan Hosseini, Roanne Thomas, Lara Pilutti, Pascal Fallavollita, Jeffrey W. Jutai

**Affiliations:** 1 School of Rehabilitation Sciences, Faculty of Health Sciences, University of Ottawa, Ottawa, Ontario, Canada; 2 Interdisciplinary School of Health Sciences, Faculty of Health Sciences, University of Ottawa, Ottawa, Ontario, Canada; 3 Brain and Mind Research Institute, University of Ottawa, Ontario, Ottawa, Canada; 4 Interdisciplinary School of Health Sciences, Faculty of Health Sciences, University of Ottawa, Ontario, Ottawa, Canada; 5 School of Electrical Engineering and Computer Science, Faculty of Engineering, University of Ottawa, Ottawa, Ontario, Canada; 6 Interdisciplinary School of Health Sciences, Faculty of Health Sciences, and Life Research Institute, University of Ottawa, Ottawa, Ontario, Canada; Tsinghua University, CHINA

## Abstract

**Background:**

Little is known about the experience and the social and contextual factors influencing the acceptance of virtual reality (VR) physical activity games among long-term care (LTC) residents. Our study aims to address this research gap by investigating the unique experience of older adults with VR games. The findings will provide valuable insights into the factors influencing VR acceptance among LTC residents and help design inclusive VR technology that meets their needs and improves physical activity (PA) and well-being.

**Objective:**

We aimed to: (1) investigate how participants experience VR exergames and the meaning they associate with their participation; and (2) examine the factors that influence the participant’s experience in VR exergames and explore how these factors affect the overall experience.

**Methods:**

We used a qualitative approach that follows the principles of the Interpretive Description methodology. Selective Optimization and Compensation (SOC) theory, Socioemotional Selectivity theory (SST) and technology acceptance models underpinned the theoretical foundations of this study. We conducted semi-structured interviews with participants. 19 Participants of a LTC were interviewed: five residents and ten tenants, aged 65 to 93 years (8 female and 7 male) and four staff members. Interviews ranged from 15 to 30 minutes and were transcribed verbatim and were analyzed using thematic analysis.

**Results:**

We identified four themes based on older adults’ responses that reflected their unique VR gaming experience, including (1) enjoyment, excitement, and the novel environment; (2) PA and motivation to exercise; (3) social connection and support; and (4) individual preferences and challenges. Three themes were developed based on the staff members’ data to capture their perspective on the factors that influence the acceptance of VR among LTC resident including (1) relevance and personalization of the games; (2) training and guidance; and (3) organizational and individual barriers.

**Conclusions:**

VR gaming experiences are enjoyable exciting, and novel for LTC residents and tenants and can provide physical, cognitive, social, and motivational benefits for them. Proper guidance and personalized programs can increase understanding and familiarity with VR, leading to a higher level of acceptance and engagement. Our findings emphasize the significance of social connection and support in promoting acceptance and enjoyment of VR gaming among older adults. Incorporating social theories of aging helps to gain a better understanding of how aging-related changes influence technology acceptance among older adults. This approach can inform the development of technology that better meets their needs and preferences.

## Introduction

The world’s population of individuals aged 65 and above is projected to reach 16% by 2050, indicating a significant increase in the number of older adults [1]. In Canada, the number of people aged 65 or older (18.8%) is growing faster than the number of children aged 0 to 14 years (15.6%) [2]. With the increasing proportion of older adults compared to other age groups, it is anticipated that there will be a corresponding growth in the population of older individuals residing in LTC facilities. Ensuring the well-being of older adults in LTC facilities is crucial, as they often exhibit lower levels of PA due to various factors [3, 4] with estimates suggesting that they spend about 75% of their waking hours being sedentary [5]. Promoting tailored PA programs that consider the specific needs and abilities of older adults can enhance social engagement and overall well-being [6].

Consistent engagement in PA has been demonstrated to enhance functional capabilities, overall health, and well-being, thereby reducing the risk of all-cause mortality, chronic diseases, and premature death among older adults [7] and VR literature has demonstrated the effectiveness of immersive and interactive VR games in promoting PA among older adults [8, 9]. However, for older adults to embrace and utilize new technology, positive experiences are crucial, highlighting the need to understand acceptance factors [10]. Further investigation is necessary to fully explore the potential of VR technology in enhancing PA and well-being among older adults in LTC, given their vulnerability to the negative effects of physical inactivity [11].

LTC residents represent a critical population for research and intervention due to their unique challenges and vulnerabilities. They often face limited mobility and physical activity opportunities, heightened prevalence of chronic conditions, increased risk of social isolation, and mental health concerns. Intersection of staffing challenges and issues related to the built environment resulted in suboptimal conditions for physical activity programs [12]. Given the aforementioned constraints faced by LTC residents, any tool that can reduce inactivity deserves further investigation [13].

The aim of this study is to investigate the social and individual factors influencing the acceptance of VR physical activity games among residents in LTC facilities. While previous qualitative research has examined the potential benefits of VR technology in promoting physical activity, well-being, and social interaction among older adults [14–16] existing studies have specific limitations that underscore the need for further research. Chaze et al. [14] focused on evaluating VR content tailored to a specific organization, limiting generalizability. Lin’s study [16] approached VR leisure activities from a marketing perspective, potentially differing from the needs of LTC residents. Kruse’s study [15] compared acceptance of VR exergames with traditional video games but did not delve into the influencing factors among older adults. Therefore, further research is needed to understand acceptance factors, inform design, and implement effective VR interventions for LTC residents, promoting PA and overall health outcomes. Our study seeks to address this gap by exploring the social determinants of VR acceptance among older adults, informed by social theories of aging. By conducting open-ended interviews and examining the factors that influence VR acceptance, we aim to inform the design and implementation of VR technology in LTC facilities, ultimately enhancing accessibility and effectiveness for this population.

The Technology Acceptance Model (TAM) [17] is commonly used to study technology acceptance but has limitations in real-life contexts [18]. This criticism highlights the need to understand the social determinants of VR acceptance among older adults, which requires research informed by social theories of aging. Open-ended interviews, as suggested by Lin et al. [16]. This research can inform the design and implementation of VR technology for older adults, enhancing accessibility and effectiveness.

Considering the identified research gaps, exploring the social determinants of VR acceptance among older adults can provide valuable insights into improving the accessibility and effectiveness of VR technology for this population. This focus is aligned with the objective and research questions of our study.

### Objectives and research questions

The primary objectives of this study are twofold:

1. To comprehensively explore the experiences of older adults with VR exergames.
2. To identify and analyze the individual and organizational factors that significantly influence their experiences and the acceptance of VR games.

To achieve these objectives, we have formulated two specific research questions:

1. How do participants experience VR exergames, and what meaning do they attach to their participation?
2. What are the factors that influence the participants’ experience, and how do these factors affect the experience?

## Theoretical framework

Our study was guided by SOC [19] theory and SST [20] as frameworks to investigate the social and contextual determinants of acceptance of VR technology. SOC focuses on older adults’ awareness of losses and gains and the decline in their resources as a consequence of aging [19]. According to this theory, individuals select more important or more attainable goals, optimize their performance to maximize the gains, and compensate unachieved goals to maintain functioning [21]. SOC theory guides our exploration of the selection and optimization of activities and the compensation for losses, while SST underscores the significance of emotionally meaningful social interactions in the context of technology acceptance. SST is a lifespan theory that helps explain the shift of personal goals and behaviors with age. SST introduces the concept of future time perspective (FTP), which refers to one’s perception of time and how much time is left [20]. The study also draws on the components from TAM [17], The Unified Theory of Acceptance and Use of Technology (UTAUT) [22], and The Senior Technology Acceptance Model (STAM) [23] to investigate the psychological factors affecting VR acceptance. By considering both psychological and social factors, we aim to gain a comprehensive understanding of VR acceptance among older adults and inform interventions and strategies for promoting VR adoption.

TAM indicates perceived usefulness (the extent to which using a specific application system will increase job performance) and perceived ease of use (the subjective probability that using technology will be free of effort) shape the attitudes of the user toward technology that affect the intention of use and determine computer acceptance behavior [17].

Venkatesh and colleagues developed UTAUT as an integration model of eight dominant theories and models with seven core determinants of intention and usage and up to four moderate determinants [24]. They stated that performance expectancy, effort expectancy, social influence, facilitating conditions, hedonic motivation, price value, and habit as direct determinants and gender, age, and experience as the key moderators play a significant role in user acceptance and usage behaviour.

The senior technology acceptance model (STAM) is the first TAM-based model that goes beyond TAM’s organizational and business context and is specifically formulated to understand gerontechnology acceptance by older adults’ predictive factors of TAM and UTAUT [23].

We recognize that while technology acceptance models focus on psychological factors to explain users’ acceptance of technology, they overlook the social processes associated with aging. Therefore, we emphasize the importance of considering both psychological and social factors to comprehensively understand VR acceptance among older adults. To fully understand VR acceptance among older adults, both psychological and social factors should be considered. This integrated approach informs interventions and strategies for promoting VR adoption and use. By uncovering social and contextual determinants, this study aims to enhance VR uptake and use among older adults, improving their overall health and well-being.

Our study integrated SOC theory and SST to tailor VR exergame interventions to older adults’ preferences, well-being, and continued participation [19, 20, 25]. SOC theory’s strategies for successful aging guide the selection and optimization of activities, compensating for losses. SST emphasizes prioritizing emotionally meaningful social interactions. Incorporating features that support social connections, emotional well-being, and cognitive and physical stimulation in VR games is crucial to fulfill older adults’ life goals.

Theoretical frameworks of aging guided our data analysis, identifying themes related to social support and goal-driven attitudes towards VR gaming. By analyzing participant interviews, we aimed to understand the role of social support and the influence of goals on attitudes towards VR games. These themes were derived from the application of social theories of aging, providing structure and direction to our study.

To gain insight into factors influencing older adults’ acceptance of VR exergames, we employed TAM components in analyzing staff perspectives. The use of TAM models helped identify key themes related to acceptance. Our study’s integrated approach to theoretical frameworks allows for a comprehensive examination of VR acceptance among older adults, addressing both psychological and social factors to enhance VR uptake and use, ultimately improving overall health and well-being in this population.

## Materials and methods

### Methodology

Our study utilized interpretive description [26] as a qualitative research methodology to understand the unique VR gaming experiences of LTC residents. This approach goes beyond traditional descriptive methods, aiming to uncover deeper meanings and explanations in specific contexts. This approach delves into deeper meanings and explanations within specific contexts. Focusing on the VR gaming experiences of LTC residents and tenants, interpretive description allowed us to explore their perspectives, challenges, and coping mechanisms. By unraveling these unique aspects, this methodology contributes to the development of targeted interventions aligned with the specific needs and preferences of LTC residents and tenants. Emphasizing contextuality ensures findings are not only insightful but also directly applicable to the intricate factors characterizing the LTC environment. In essence, our choice of interpretive description underscores our commitment to understanding the depth and richness of participants’ VR gaming experiences, with direct implications for enhancing their well-being within clinical and care settings. Through semi-structured interviews with residents, tenants, and staff members, we gained practical insights and theoretical understanding by exploring characteristics, patterns, and structures. By employing deductive reasoning, we identified initial codes, themes, and concepts derived from existing theories and models. This study was conducted with ethical approval from the University of Ottawa’s Health Sciences and Sciences Research Ethics Board (Ethics File Number: H-02-22-7627).

### Participant recruitment

Data were collected from July 4, 2022 to August 10, 2022 at a community healthcare center serving older adults and veterans in long-term care homes and independent apartments. The facility accommodates two types of individuals: residents and tenants. Residents, typically elderly adults, require round-the-clock care and assistance with daily tasks. On the other hand, tenants sign lease agreements with the facility for a specific duration and enjoy greater autonomy. The participants in this study were a subsample from a related study on VR games conducted a few days prior to the interviews.

Residents and tenants who met the following inclusion criteria were selected for participation: (1) aged 65 or older, residing in LTC; (2) previously took part in the VR games study [27]; and (3) able to communicate and read in English. Written informed consent was obtained from those who agreed to participate before the interviews (See S1 File).

Staff members who met the following criteria were included: (1) employed at the facility for at least six months; (2) proficient in English; and (3) directly involved in resident care. Exclusions included staff with less than six months of employment, limited English proficiency, or no direct experience with residents. Gathering staff perspectives provided a comprehensive understanding of the study topic given their close proximity and extensive knowledge. Purposive sampling involved sending emails to staff members responsible for resident activities and programs. The first four staff members who agreed to participate were selected and signed written informed consent before the interviews (See S2 File). This sampling approach ensured the selection of staff members most suitable to provide insights into the experiences of older adults and veterans in LTC facilities and independent apartments.

In our VR games study, participants engaged with ***Song Beater***, a VR rhythm game that challenges players’ sense of rhythm through dance and slashing flying beats [28]. Despite not being specifically designed for older adults, Song Beater offers several features that make it suitable for this population, including customizable difficulty levels, seated playing option, one-handed playing with balance support, customizable environment, and the ability to use personal music and adjust song speed. Participants played the game three times within a two-week period.

### Data collection

Semi-structured interviews were conducted in person between July and August 2022, within three days after the VR gaming study. Prior to the interviews, participants were provided with an information letter and asked to sign consent forms, ensuring the confidentiality of their responses. All participants were asked the same set of questions regarding their experience with the VR game.

Participants were interviewed once, within 3 days after their participation in the VR gaming study, allowing for sufficient reflection time and reducing initial biases or immediate reactions. Interviews took place in participants’ apartments or private rooms at the LTC facility to ensure comfort and ease during the process. The interviews lasted 15–30 minutes, respecting participants’ time and attention spans while gathering meaningful information. A set of nine open-ended questions, focusing on game characteristics, preferences, experiences, and goals, were used (See S3 File). interview questions were designed to be open-ended and neutral to allow participants to express their experiences and opinions freely without being influenced by the researchers’ preconceptions. Probing questions and follow-up prompts were employed to elicit detailed responses and ensure comprehensive coverage of relevant aspects [29]. Interview questions were structured to explore themes related to the theoretical framework’s constructs, ensuring that participant experiences and attitudes towards VR gaming were examined through the lens of age-related variations and social determinants. For instance, questions were designed to probe participants’ interests, goals, social interactions, emotional experiences, and perceptions of game characteristics, aligning with the concepts outlined in SOC, SST, and TAM.

Staff members were interviewed after resident data collection to gather potential feedback. They received information letters, provided informed consent, and answered nine open-ended questions (See S4 File) regarding barriers to physical activity and factors influencing technology acceptance by LTC residents. Interviews took place during staff members’ break time in their offices, lasting 15–30 minutes to minimize work disruption.

### Data analysis

Interviews were audio-recorded and transcribed verbatim. The data analysis followed the interpretive description methodology [21] and involved three stages: data familiarization, theme and pattern identification, and theme labeling and reporting. NVivo 12 qualitative analytic software (QSR International, release version 1.6.1) was used for coding the transcripts. Inter-rater reliability [30] was ensured through independent coding of five transcripts (three of the transcripts were the same and two were different) by two researchers, followed by consensus discussions to address any discrepancies. Inter-rater reliability checks were conducted to ensure consistency and objectivity in the data analysis process and reduce the risk of individual biases shaping the interpretation of findings. As analysis progressed, the theme "game characteristics" was refined into "Enjoyment, excitement, and the environment" to capture a nuanced understanding of player experiences.

Preliminary themes were developed through detailed analysis and discussions, considering the source data and codes [31]. Observations and field notes provided valuable context. The data were examined for themes related to TAM, SOC, and SST constructs, providing a theoretical foundation for the study. A combination of social and psychological theories was employed to explore technology attitudes and their age-related variations. SOC guided the exploration of participants’ interests, goals, and social interactions, while SST focused on emotional experiences and social connections. TAM facilitated the examination of game characteristics, perceived usefulness, and ease of use.

To maintain the integrity of our study, we followed established qualitative research principles, employing interpretive description as our robust methodology to explore the distinct VR gaming encounters of LTC residents. Clear participant recruitment criteria, ethical approval, and informed consent were meticulously addressed to enhance credibility and respect ethical considerations. A strategic sampling approach, detailed VR game description, and structured data collection with temporal considerations were implemented to provide a comprehensive understanding of the study topic. Rigorous data analysis using NVivo 12 software, inter-rater reliability, and a theoretical foundation grounded in social and psychological theories further strengthened the study’s credibility, dependability, and transferability. These methodological considerations collectively contribute to the rigour of our study and formed a solid foundation for meaningful interpretation and generalization of findings.

## Result

### Resident and tenant participants’ characteristics

Twelve tenants (80%), three residents (20%), and four staff members from an LTC facility participated in the interviews. The participants’ ages ranged from 66 to 93, with a median age of 78. The gender distribution was eight female participants (53.3%) and seven male participants (46.7%). The number of participants was above the recommended number for saturation by Guest and colleagues [32], as which suggests that data saturation occurs within the first twelve interviews in qualitative methods. Educational backgrounds varied, with 40% having a bachelor’s degree or higher. Most participants (66.7%) had prior gaming experience, while the remaining (n = 5, 33.3%) did not. Table 1 depicts resident and tenant participants’ characteristics.

**Table 1:** Resident and tenant participants’ characteristics.

Characteristics	No. (total = 15)	% (100)
Age (y)		
93	1	6.7%
78	3	20%
82	2	13.3%
75	1	6.7%
84	1	6.7%
70	1	6.7%
81	1	6.7%
77	1	6.7%
66	1	6.7%
83	1	6.7%
85	1	6.7%
69	1	6.7%
Gender		
Female	8	53.3%
Male	7	46.7%
Education level		
High school diploma	2	13.3%
Apprenticeship or other trades certificate	3	20%
College diploma	2	13.3%
University below bachelor’s	2	13.5%
Bachelor’s degree or higher	6	40%
Live in		
Long-term care apartments	3	20%
Tenant apartments	12	80%
Prior gaming experience		
Yes	10	66.7%
No	5	33.3%

We interviewed four staff members (two women and two men). They had varying years of experience in the facility from 1–10 years. Three were health professionals while the fourth was a recreation specialist.

### Identified themes

We developed four themes to understand older adults’ VR experience and perceptions: (1) enjoyment, excitement, and the novel environment; (2) PA and motivation to exercise; (3) social connection and support; and (4) individual preferences and Challenges. In addition, themes were identified from staff members’ data, including (1) relevance and personalization of the games; (2) training and guidance; and (3) organizational and individual barriers.

Themes developed from older adults’ data were analyzed using SOC and SST. These themes relate to experience of VR gaming and how older adults prioritize certain activities and social connections as they age and compensate for declines in other areas and how they prioritize emotionally meaningful goals.

Staff members’ themes of “Relevance and personalization,” “Training and guidance,” and “Organizational and individual barriers” were analyzed using TAM, STAM, and UTAUT. These themes relate to the perceived usefulness and ease of use of the technology, as well as individual and organizational factors that may facilitate or hinder its acceptance.

The themes were derived through iterative coding and thematic analysis, where recurring patterns and insights emerged from the participants’ narratives. For example, the theme of "Enjoyment, excitement, and the novel environment" emerged from participants’ reflections on their experiences with VR gaming. Constructs such as enjoyment, excitement, and immersion were identified through careful analysis of participant responses, highlighting their emotional and experiential dimensions.

Similarly, the theme of "Physical activity and motivation to exercise" reflected participants’ perspectives on the role of VR exergaming in promoting physical activity and motivation. Constructs like physical exertion, motivation, and exercise adherence were inferred from participant accounts, demonstrating the multifaceted impact of VR gaming on physical well-being.

These themes were analyzed in the context of theoretical frameworks such as SOC, SST, TAM, STAM, and UTAUT, which provided a theoretical lens to interpret the findings. Constructs from these frameworks were mapped onto the identified themes, allowing for a deeper understanding of the underlying mechanisms driving participants’ experiences and perceptions.

### Older adults’ themes- The meanings attached to the VR gaming experience

These themes represent the experience of VR exergaming and portray the meanings that older adult participants attached to their VR gaming experience. Table 2 shows an example of how questions, themes and constructs of theoretical frameworks are formed and connected in older adults’ themes.

**Table 2:** Older adults’ themes examples.

Question	Theme	Description	Quotes	Theory
How did you feel when you were playing the game?	enjoyment, excitement, and the novel environment	Refers to the participants’ feeling when they played with the game and any other quotes about their feelings included in their responses	I felt so excited! It was fun! It’s really neat! It just brings happiness" (P5). "I liked the environment; it was very beautiful, the underwater environment"	optimized the enjoyment by engaging in VR game, compensating for any physical limitations (SOC). maximize enjoyment and positive emotions (SST)
Does playing a game like this make you feel like you are exercising/ moving?	Physical activity and motivation to exercise	Refers to the quotes about the physical activity aspect of the game and motivation are coded as physical activity	"For my age, it’s a good way to exercise, for the muscle and for the visuality" (P9). “I think the challenge of getting as highest score as you possibly can, so you do it once, you get a score, the second time you want to beat your score.” (P4)	sense of purpose, motivation to improve scores and challenge themselves, and increased self-awareness (SOC) choose goals and activities that provide personal satisfaction while minimizing effort (SST)

#### Enjoyment, excitement, and the novel environment

This theme represents older adults’ reflections on the pleasure, enthusiasm, and sense of novelty derived from engaging in VR gaming. Participants in the VR gaming experience found themselves immersed in a captivating world, filled with excitement, vibrant environments, and joyous interactions. As one participant put it, "*I felt so excited*! *It was fun*! *It’s really neat*! *It just brings happiness" (P5)*.

Participants found the VR gaming experience enjoyable, with the most cited features being the novel environment and fun nature of the game. Three participants specifically identified their gaming experience as fun, with one stating, *"Look at the beautiful fish"* (P5), another expressing, *"Neat*, *awesome"* (P14), and a third participant exclaiming, *"I saw a cat*! *I thought it was my cat*!*"* (P12). They continually commented on the environment and game details and laughed when surprised during gameplay. One participant expressed their overall enjoyment, stating, *"It’s been a fun experience which I am thankful for"* (P11).

Participants emphasized the importance of various game characteristics. Vibrant colors, immersive environments, and high-speed dynamics were key factors contributing to their enjoyment. The realistic design elements, such as Egyptian pyramids and underwater worlds, along with animated animals and fish, further enhanced the gaming experience. One participant appreciated the visually stunning environment, stating, *"I liked the environment; it was very beautiful*, *the underwater environment"* (P11). Another participant expressed fascination with dynamic elements in the background, particularly observing animals like the lion, which added to their immersion: *"Something that I found interesting was in the background*. *The animals and the other things going on and watching the big cat*, *the lion*…*"* (P6). These insights provided a deeper understanding of the specific game characteristics that captivated and brought enjoyment to the participants.

The VR game’s fast and seamless responsiveness to participants’ movements was surprising, providing an empowering and liberating experience, especially for those with physical limitations. One participant expressed their astonishment, stating, *"How crazy is that in this world*? *You are not vulnerable even*, *the VR*, *move a piece*, *and it happens right away*!*"* (P5). This quote suggests that VR allows individuals to freely engage in activities without feeling vulnerable, a possibility that may not exist in the physical world.

The game provided a unique and engaging experience, capturing participants’ attention and interest throughout gameplay. It instilled a sense of accomplishment and satisfaction as they progressed. One participant described it as follows: *"It was very engaging*… *it built intensity*… *it wasn’t just one thing around every minute*. *It slowly introduced maneuvers*, *picked up the speed*, *and became very challenging"* (P4).

Many participants appreciated the immersive nature of VR technology, which enabled them to fully engage in the virtual game world and interact with it realistically and intuitively. As one participant expressed, *"I feel like I’m facing real life*, *but the design is not quiet*. *I want to be immersed more in the game"* (P9).

Music played a crucial role in generating excitement during the game. Participants actively paid attention to the songs and recognized their impact on the game’s rhythm and flow. Some even sought out other songs from the game’s playlist to increase the pace and excitement. Perspectives on the role of game music varied among participants. One participant expressed deep appreciation, explaining how it enhanced their personal involvement and enjoyment: "*I love music; I love dancing*, *so then it brought more of me with the music*. *The music*, *I would say*, *I’m present more*. *[Laugh]"* (P7). In contrast, another participant viewed game music as mere auditory disturbance, describing it as *"Music is a noise"* (P11).

Such findings align with the framework of SOC which proposes that individuals adapt to the aging by focusing on their goals and optimize the meaningful activities while compensating for the limitations. Participants optimized the enjoyment by engaging in VR game, compensating for any physical limitations. The findings align with the SST framework. Participants in this study sought out VR gaming to maximize enjoyment and positive emotions. The SST lens highlights how older adults are driven by emotional motivations, seeking enjoyable experiences like VR gaming to foster positive emotions and enjoyment throughout the aging process.

#### Physical activity and motivation to exercise

This theme explores older adults’ perspectives on the role of VR exergaming in promoting PA, motivation, and adherence to exercise routines. Participants recognized the connection between PA and motivation during their VR gaming experience. They acknowledged the importance of PA within the VR context, which provided benefits such as improved coordination, balance, and upper body workout. Two participants highlighted the positive exercise effects; P4 stated, *"I have some exercise from it*, *and I’m conscious of that*, *as my arms go back and forward and try to catch those Frisbees"* (P4). Similarly, P9 described the game as "a good way to exercise" and emphasized the muscular and visual benefits: *"For my age*, *it’s a good way to exercise*, *for the muscle and for the visuality"* (P9).

Another participant mentioned feeling warm during the gameplay and needing to turn on a fan, suggesting that the game provided a demanding PA that raised their body temperature. However, some participants viewed the game as an *"add-on"* (P2) or an *"upper body exercise"* (P8), focusing primarily on engaging the upper body rather than the entire body.

In addition to physical benefits, Participating in the VR game also brought awareness to some older adults regarding their physical limitations. One participant expressed frustration with their left hand’s accuracy compared to their right hand, stating, *"I was annoyed with myself that my left hand was not as accurate as my right hand*. *I’ve always been right-handed*. *I don’t use my left hand very much at all"* (P8). This realization showcases how VR has the potential to enhance self-awareness and uncover previously unnoticed physical limitations.

Besides the physical benefits, the challenge of the game and the sense of accomplishment that came with overcoming it motivated the players to perform better, achieve higher scores, and test their abilities. Some players even saw it as a challenge against the VR machine and tried to outsmart the system. As one participant stated: *“I think the challenge of getting as highest score as you possibly can*, *so you do it once*, *you get a score*, *the second time you want to beat your score*.*” (P4)*

A participant expressed that the game served as motivation for PA and helped them escape daily life challenges, offering a distraction and alleviating depression, *"It takes me out of my everyday tasks and everything and brings me to another world… you see the fishes and things like that and makes you forget you are in the house*, *and you take the thing [headset] out*, *and you see oh*! *I’m in the house"* (P12). Another participant emphasized the importance of the VR experience in combating social isolation and building confidence post-COVID-19 lockdowns. The VR experience brought them happiness and a sense of security, preparing them for rejoining society. *“A really good space to be in*, *after you have a bad day*, *wow*! *Increase your thoughts and your endorphins*, *you know*, *just puts you in a happy mood*.*” (P5)*

The use of SOC revealed that VR gaming gave older adults a sense of purpose, motivating them to improve scores and challenge themselves. It also increased self-awareness, as one participant discovered accuracy differences between their hands. VR gaming contributed to physical well-being by providing a meaningful activity that enhanced self-awareness and personal growth.

Also, SST helps us understand the motivation aspect of the theme. According to SST, individuals choose goals and activities that provide personal satisfaction while minimizing effort. In this case, VR gaming offered a fun way for older adults to be physically active, providing a sense of accomplishment and motivation. It motivated participants to exercise, bringing happiness and well-being by serving as a distraction from daily challenges.

#### Social connection and support

This theme refers to older adults’ experiences of social interactions, connection, and emotional support facilitated through VR gaming, including the impact on social relationships. Participants viewed the game as an opportunity to socialize and connect with others, whether with their friends in the facility or family members who live far away: *“I wouldn’t mind playing my grandson*. *[It] Would be a challenge*, *if I wasn’t successful that was fine*, *but this is something to do together*.*”* (P7) One participant expressed their preference regarding a possible setup for the game for group gameplaying: *“If it could be set up for two players and one hit one colour*, *and one hit the other colour*, *that would be a challenge”* (P10).

Participants went beyond involvement, recommending friends to the researcher as potential participants. Their proactive approach and follow-up inquiries demonstrated interest in the study’s success and created a sense of community. This highlighted the importance of social support in promoting engagement and participation, showcasing the potential benefits of VR gaming for LTC facility residents.

Participant sharing of VR gaming experiences enhanced self-efficacy and performance, highlighting the potential of social support and knowledge sharing. Technical support increased confidence in technology use, with a desire to continue playing if volunteer assistance is available: *"If we have a volunteer*, *hopefully*, *we can*. *Somebody that could be available"* (P5). Two participants emphasized the importance of support in navigating the game: *"You escorted me through*… *if I had been there by myself*, *I might have had difficulties"* (P4), *"If you weren’t here*, *I don’t know how I would go through"* (P5).

Staff and family encouragement significantly influenced acceptance and enjoyment of VR games. One couple shared their scores with their son, who expressed amazement and motivated them to continue: *"I certainly would be interested in another project*… *I said to my son*: *I tried that [VR]*, *and I guess I was pretty good at"* (P7).

Participants were motivated to engage in VR gaming for socialization, aligning with SOC principles. SST emphasizes prioritizing emotionally meaningful relationships as individuals age. Participants’ enthusiasm and support for the VR gaming study highlight potential benefits for LTC residents and the importance of social support in promoting engagement. Technical assistance during the VR gaming experience was crucial, emphasizing the role of emotional support and guidance.

#### Individual preferences and challenges

This theme explores participants’ preferences, challenges, and barriers in VR exergaming, including technical difficulties, physical limitations, and personal preferences for game features. Participants’ experiences were influenced by their interests and prior gaming experience. Some expressed enthusiasm to explore the full potential of VR: *"I would like to continue with VR and explore its capacity"* (P3, experienced gamer). Others, with less experience or health concerns, appreciated the exercise and novelty: *"Good exercise*, *concentration*, *focus*, *and challenge"* (P14, with comorbidities).

The gaming experience had some challenges for participants. Those without prior experience found the game setup and menu navigation difficult, hindering their engagement. Some participants, even after adjusting the speed, felt the game was too fast for their reflexes: *"Perhaps for an older person*, *it must be slower"* (P7). These challenges impacted their enjoyment and acceptance of the game.

The boxing game involved punching flying objects without button presses, but the controller buttons posed challenges for some participants. Accidental button presses interrupted the game, requiring extra effort to resume: *“I was happy to see how easy it was*, *except about the controllers*. *I never got master of those*.*”* (P7) Additionally, some participants found the headset weight burdensome, further affecting their experience and interaction with the game.

Some participants prioritized health-related goals to maintain an active and enjoyable life: *"to keep a healthy and active life"* (P1) and *"to reach 100"* (P5) to spend time with grandchildren. The goals expressed by participants align with the SOC theory, as they actively select and optimize important goals while compensating for age-related limitations. These goals include maintaining physical activity, controlling blood sugar, and losing weight, reflecting their focus on health and well-being.

The participants in our study actively selected and optimized health-related goals, such as maintaining physical activity and controlling blood sugar, while compensating for age-related limitations, aligning with the SOC theory. These goals were emotionally meaningful, contributing to their overall quality of life. Additionally, goals related to family time and fostering social connections within their living facility were consistent with the SST, highlighting the importance of social relationships and emotional well-being.

### Staff members’ themes- Determinants of VR acceptance by residents

The staff members’ perspectives provided valuable insights into the factors influencing the acceptance of VR games among LTC residents. Being in frequent communication with the residents and involved in decision-making regarding recreational programs and physiotherapy practices, the staff members were well aware of the residents’ needs and preferences. Table 3 depicts examples of the questions, themes, quotes and relevant models.

**Table 3:** Staff members’ themes examples.

Question	Theme	Description	Quotes	Theory
What are the factors that you think may affect the acceptance of VR among residents?	Relevance and personalization	Refers to the staff members’ quotes about the importance of relevant and personalized content in acceptance of VR games by older adults	*Having whatever they see relevant to their lives may affect them here"* (S1).	TAM’s perceived usefulness
What are the factors that you think may affect the implementation of VR at the facility?	Organizational and individual barriers	Refers to the quotes about the organizational and individual barriers and facilitators identified by staff members	"How much does it cost for that technology? And then would the facility be able to buy it?" (S1). "If it takes a long time to set up. . . whatever it is… But also troubleshooting. Because if there is a problem with it, I know the healthcare team doesn’t have any time to really do it" (S1).	Price value (ATUAT) Facilitating conditions (ATUAT)

#### Relevance and personalization

Staff members stress the importance of tailoring VR games to LTC residents’ individual needs and interests for better acceptance. This involves designing games with relevant themes and user-friendly equipment. One staff member stated, "*Having whatever they see relevant to their lives may affect them here"* (S1). Participants’ experiences support this approach, with one likening the game to the View-Master and another recalling positive memories while playing. VR gaming has the potential to evoke positive emotions and increase acceptance among older adults.

The relevance and personalization of VR games can be analyzed using TAM’s perceived usefulness component. TAM highlights that perceived usefulness and ease of use determine technology acceptance. When games align with players’ interests, they are seen as more useful, increasing acceptance. Additionally, user-friendly equipment enhances ease of use, further promoting acceptance.

#### Training and guidance

Staff members highlighted the role that education and support from both family members and staff members play in the acceptance of VR games among residents. They emphasized the importance of educating LTC residents on the proper use and potential benefits of VR games. This education can help increase residents’ understanding and familiarity with the technology, leading to higher acceptance and engagement.

They believed that family members could provide emotional support and act as a source of guidance and information, helping residents feel more comfortable and confident using VR games *“If you have a family that is very supportive of this project and trying this new thing*, *it would play a huge role*.*”* (P3). On the other hand, staff members can provide hands-on assistance and support during VR games, helping residents navigate the technology and troubleshoot any issues that may arise:

A staff member emphasized the need for researchers and staff to prioritize relationship-building when working with LTC facility residents, especially in research or implementing new technologies like VR. Due to the residents’ vulnerability and unique perspectives, it is crucial to consider their specific needs. Older adults who are introduced to VR require trust and a sense of safety to fully embrace the new experience. This can be established through effective communication and comprehensive training provided by researchers: *“Taking that time to build those relationships because using a headset it’s like*, *virtual reality*, *it changes your reality*, *so you need to know that you are safe*, *somebody safe is there with you*.*” (S4)*

Staff members believed that educating LTC residents on the benefits of VR games and providing them with guidance can help increase their understanding and familiarity with the technology, leading to higher acceptance and engagement: *“It’s just really a lot about education*.*”* (P4). This aligns with the TAM model’s emphasis on perceived usefulness as a key factor in technology acceptance.

#### Organizational and individual barriers

Staff members noted organizational and individual challenges that hinder the acceptance of VR games among LTC residents. These barriers, identified through interviews, can be categorized as affordability, equipment maintenance, and a shortage of staff to assist with VR headset usage and the individual barrier relates to health issues.

One significant organizational barrier is the cost of purchasing VR sets, which can limit availability and accessibility for LTC residents: *"How much does it cost for that technology*? *And then would the facility be able to buy it*?*"* (S1). Troubleshooting, equipment maintenance, and a shortage of staff assistance further hinder the implementation of VR gaming in LTC settings. Technical issues require specialized knowledge, and the limited availability of trained staff to assist residents with VR headset usage poses challenges: *"If it takes a long time to set up*… *whatever it is… But also troubleshooting*. *Because if there is a problem with it*, *I know the healthcare team doesn’t have any time to really do it"* (S1).

Individual barriers encompass cognitive and physical health issues faced by LTC residents. Cognitive impairments, such as dementia or age-related decline, can hinder their comprehension and engagement with VR gaming: *"There are barriers with Dementia*… *your perception of reality might be disordered"* (S3). Mobility limitations can also impact their ability to interact with VR equipment and participate in physical activities within the virtual environment: *"Maybe it’s a ball game asking to the left*, *and the physical problem would be on the left arm*. *So*, *the difficulty would be how to reach the ball on time"* (S2).

The mentioned organizational barriers, including affordability, difficulties with equipment maintenance, and staff shortage, can be associated with the UTAUT model’s construct of price value and facilitating conditions [22]. Individual barriers, such as physical and cognitive health issues, can be linked to the STAM model’s constructs of physical functioning and cognitive ability [23].

## Discussion

Our study offered unique insights that advance the existing literature on VR technology in LTC settings. By drawing upon social theories of aging, such as SOC and SST, we gained a deeper understanding of how older adults prioritize activities and social connections while navigating declines in other areas. This theoretical grounding allowed us to explore the motivations and barriers influencing technology acceptance among older adults, providing a comprehensive understanding of VR technology acceptance in LTC facilities.

One of the significant findings was the multifaceted benefits of VR gaming experiences for older adults, including physical, cognitive, social, and motivational aspects. These benefits suggest that VR technology has the potential to enhance the lives of older adults in LTC facilities by providing new opportunities for engagement and interaction. For instance, the immersive nature of VR offered an escape from pandemic-related stress and isolation, thereby improving social interactions and cognitive stimulation.

Our study also identified key factors influencing the acceptance of VR technology, such as the importance of staff understanding residents’ needs, education and support from family and staff, and addressing organizational barriers like cost and maintenance. These findings underscore the necessity of a supportive environment for successful VR implementation.

Varying levels of technological familiarity among participants and different perspectives between older adults and staff members were the challenges in interpreting the findings of our study. Older adults’ experiences were influenced by their emotional and social priorities, while staff members focused on practical considerations like perceived usefulness and ease of use.

Our findings align with existing literature on VR’s impact on social engagement among older adults in LTC settings. Factors like staff training, and barriers such as technology adaptability and concerns about technology characteristics, reflect common themes in prior research [33]. Recognizing these parallels underscores VR’s potential to enhance social engagement and well-being in LTC. The findings of our study indicated that residents and tenants of LTC facilities can derive physical, cognitive, social, and motivational benefits from VR gaming experiences. This aligns with previous research by Peng et al. [8] and Skjæret et al. [9] highlighting the positive impact of VR games on facilitating physical activity and overcoming space or staffing limitations. Importantly, our study demonstrated that older adults could enjoy and adapt to VR games with proper guidance.

Our research underscored the significance of understanding older adults’ needs and preferences in designing personalized VR programs. Similar to studies by Chaze et al. [14] and Lin et al. [16], our emphasis on ensuring a positive experience with VR technology highlighted the importance of fun, safety, and ease of engagement. Emotional and hands-on support, education on proper use and benefits of VR games, and establishing trust and safety emerged as critical factors for increasing acceptance and engagement among older adults.

In conclusion, our study successfully investigated older adults’ attitudes towards a VR physical activity game and identified key factors influencing their acceptance of this technology. By focusing on individual and social aspects, we provided insights into participants’ perceptions of VR technology’s usefulness, immersion experiences, and feelings of comfort and safety.

We highlighted the potential of VR technology in addressing mental health challenges associated with social isolation, particularly during the pandemic. The immersive nature of VR offered an escape from stress and isolation, potentially preventing related mental health issues.

Our research also revealed distinctions between the perspectives of older adults and staff members. Older adults’ views were rooted in social theories of aging (SOC and SST), while staff members’ themes aligned with TAM models. This gap underscores the need for incorporating social theories of aging into technology studies to better understand how aging influences technology acceptance among older adults.

In summary, our study contributes to the existing knowledge on VR technology acceptance among older adults and emphasizes the importance of integrating social theories of aging in future research to address their unique needs and preferences effectively.

### Limitations and strengths

Our study addressed the gap in research on VR technology acceptance among older adults in LTC facilities. We examined both individual and social factors, uncovering dimensions not fully accounted for in existing acceptance models. Additionally, we explored the effects of VR on PA and social interactions, revealing the potential benefits of integrating VR into their daily routines.

Our study has certain limitations that need to be acknowledged. The use of masks during data collection, necessitated by the COVID-19 pandemic, posed challenges for participants with hearing issues, potentially affecting the quality and validity of the data. The prolonged lockdowns and communication restrictions could have influenced participants’ experiences and priorities, warranting consideration of the pandemic’s impact. Furthermore, the study’s focus on specific VR content restricted the generalizability of conclusions regarding VR technology acceptance in the broader population.

### Recommendations

This study contributes to the growing body of research on the use of VR in LTC facilities and provides practical implications for future research and implementation.

#### Recommendations for future research

Future investigations concerning technology acceptance among older adults should strategically integrate social theories of aging, notably SOC and SST. This methodological approach will facilitate a more comprehensive understanding of the multifaceted factors influencing the acceptance of technology among older adults. Analyzing how aging influences their acceptance through experiential lenses can yield nuanced insights, shaping the development of technology tailored to their distinct needs.

To successfully integrate VR gaming into the lives of LTC residents and comprehend the intricate interaction dynamics between older adults and VR games, researchers should explore several key areas. Investigating the enduring effects of VR gaming on physical activity, motivation, and mental health can provide a holistic assessment of its impact on the well-being of older adults. Delving into the influence of diverse music types on gameplay and user enjoyment can contribute to the creation of more personalized and engaging experiences. Additionally, exploring the potential of VR gaming as a form of physical therapy and exercise can enhance self-awareness of physical limitations.

The significance of social support in fostering engagement and acceptance of VR gaming among LTC residents is evident from our observations. Future studies should meticulously incorporate social support mechanisms in their design and execution to optimize the benefits for this demographic. Crafting personalized and relevant VR games aligned with LTC residents’ interests is paramount. Educating and supporting both residents and their families to enhance acceptance and engagement with VR games should be a central focus. Establishing trust and safety when introducing new technologies and addressing organizational and individual barriers are imperative considerations.

Lastly, research endeavors should delve into the potential of VR gaming as a tool to alleviate social isolation, a pervasive concern for many older adults. Comprehensive exploration in these areas can contribute to the effective implementation of VR gaming in LTC facilities and elevate the quality of life for older adults.

#### Recommendations for designers

In order to optimize the usability of VR games for older adults, designers are encouraged to integrate specific design principles. Firstly, an emphasis should be placed on cultivating a simple and intuitive interface, characterized by easy-to-navigate menus, larger buttons and text, and explicit instructions. This adherence to a minimalist design philosophy serves to enhance the overall user-friendliness of the interface. Secondly, the gameplay should adopt a deliberately slow-paced structure, incorporating extended intervals for each task to effectively mitigate the occurrence of motion sickness while concurrently heightening the enjoyment factor. Thirdly, designers are advised to judiciously restrict the number of head and body movements required, given the potential challenges older adults may encounter with swift or extensive motions. This strategic reduction in movement requirements stands to substantially augment the accessibility of the game. Finally, the provision of customizable settings, encompassing adjustable parameters such as brightness, contrast, speed, and text/button size, affords older adults the opportunity to tailor the game to align with their specific needs and preferences. The assimilation of these meticulous design principles is posited to empower VR game designers in the creation of games that not only exhibit heightened usability but also elicit a more enjoyable experience for the older adult demographic.

## Supporting information

S1 File Older adults interview consent form. (DOCX)

S2 File Staff members interview consent form. (DOCX)

S3 File Older adults interview guide. (DOCX)

S4 File Staff members interview guide. (DOCX)
