# Supplementary material for: Acceptance of physical activity virtual reality games by residents of long-term care facilities: A qualitative study
Source: PLoS One. 2024 Jun 25;19(6):e0305865. doi: 10.1371/journal.pone.0305865 (PMC11198859; doi:10.1371/journal.pone.0305865)
Supplement: S2 File — (DOCX) [file pone.0305865.s002.docx]

## S2 File

STAFF MEMBERS INTERVIEW CONSENT FORM

**Study Title: A Study on Identifying the Factors Associated with Seniors’ Acceptance of Virtual Reality Games**

**Understanding of my rights in research**

**Please circle YES or NO**

|  |  |  |  |  |
| --- | --- | --- | --- | --- |
| I read the letter about this study. | | | **YES** | **NO** |
| I decided that I want to be in this study. | | | **YES** | **NO** |
| I know that I do not need to be in this study if I do not want to. | | | **YES** | **NO** |
| I will participate in this study which includes one 30-minute interview. | | | **YES** | **NO** |
| I can stop my participation when I want. | | | **YES** | **NO** |
| It is okay for the researchers to use my answers when they tell people about their research. | | | **YES** | **NO** |
| I know that the researchers will not tell anybody my name. | | | **YES** | **NO** |
| It is okay for the researchers to ask me again if I want to continue participating in the study. | | | **YES** | **NO** |

**Participants:**

I had an opportunity to discuss this study, and any questions that I have asked were answered to my satisfaction. I voluntarily consent to participate in the **interview phase** of the study “**Identifying the factors associated with seniors’ acceptance of virtual reality games**”. I understand that this study is independent from The Perley Health and that refusal to participate will have no effect on my work at the Centre. I understand that I will receive a signed copy of this form.

___________________ ____________________ ___________________

Participant’s Name (Print) Participant’s Signature Date

By marking my initials here, _______, I agree to allow the researchers to contact me at a later time if they would like me to clarify any information.

**Person obtaining consent:**

I have discussed this study in detail with the participant. I believe the participant understands what is involved in this study.

_______________________ ______________________ ________________

Researcher’s Name (Print) Researcher’s Signature Date

If you have any questions regarding the ethical conduct of this study, you may contact the Protocol Officer for Ethics in Research, University of Ottawa, Tabaret Hall, 550 Cumberland Street, Room 154, Ottawa, ON K1N 6N5, Tel.: (613) 562-5387, Email: ethics@uottawa.ca).
