# Supplementary material for: Acceptance of physical activity virtual reality games by residents of long-term care facilities: A qualitative study
Source: PLoS One. 2024 Jun 25;19(6):e0305865. doi: 10.1371/journal.pone.0305865 (PMC11198859; doi:10.1371/journal.pone.0305865)
Supplement: S3 File — (DOCX) [file pone.0305865.s003.docx]

## S3 File

OLDER ADULTS INTERVIEW GUIDE

**Interview Script**

**Background and purpose**

Thank you for agreeing to participate in this interview for my thesis project on the acceptance of VR technology among residents of LTC facilities. Through this research, I explore the factors associated with the acceptance of new technologies to better understand the interaction between this group and technology.

This interview should take 30 minutes and will be audio-recorded so that I can more easily review the notes afterward.

**General questions**

- What do you think of the VR exercise game?
- How did you feel when you were playing the game?

**Game preference questions**

- What do you like about the game you played?
- What do you dislike about the game you played?
- Is this a game that you would like to continue playing?
- What is interesting about this game that would make you continue playing?
  - **If they said No:** How can we make it better/more interesting for you?
- How do you prefer to play? (Alone or with others)
- Does playing a game like this make you feel like you are exercising/ moving?
  - **If they said No:** Is it something about the game, your condition, …?
- Prob questions: What stops you? Can you tell me? Why do you say that?

At this point, let me ask you do you want to add something? I have one more question to ask you. Let’s talk about what kind of goals you have set goals for yourself. Do you have goals for yourself? If the answer is NO, the prob question would be, what about your health? Do you have hobbies? How about other activities and hobbies? E.g. doing puzzles, book clubs, knitting, and visiting grandchildren?

Thank you so much for your time. Sharing your experience and thoughts on VR gaming will help us to reach a better understanding of technology acceptance by seniors.
