# Supplementary material for: Acceptance of physical activity virtual reality games by residents of long-term care facilities: A qualitative study
Source: PLoS One. 2024 Jun 25;19(6):e0305865. doi: 10.1371/journal.pone.0305865 (PMC11198859; doi:10.1371/journal.pone.0305865)
Supplement: S4 File — (DOCX) [file pone.0305865.s004.docx]

## S4 File

STAFF MEMBERS INTERVIEW GUIDE

1. Please briefly introduce yourself and your responsibility at this facility.
2. How often do you meet with residents?
3. Do you have any responsibility for making decisions for recreational and therapeutic activities or programming for residents? Yes: describe
4. Are you familiar with VR technology?
5. Yes: Please describe.
6. Do you think VR could be a good way to encourage residents to exercise?

What would you say are the potential benefits of VR tech for residents?

What do you think are the potential barriers to using VR technology with residents?

1. What are the factors that you think may affect the acceptance of VR among residents?
2. What are the factors that you think may affect the implementation of VR at the facility?
3. Are there any other things that you would like to tell me that might help our understanding of VR with this population?
